# The Evolutionary History of Daphniid *α*-Carbonic Anhydrase within Animalia

**DOI:** 10.1155/2015/538918

**Published:** 2015-03-29

**Authors:** Billy W. Culver, Philip K. Morton

**Affiliations:** ^1^Program in Ecology & Evolutionary Biology, Department of Biology, University of Oklahoma, 730 Van Vleet Oval, Norman, OK 73019, USA; ^2^University of Oklahoma Biological Station, 15389 Station Road, Kingston, OK 73439, USA; ^3^Murray State College, One Murray Campus, Tishomingo, OK 73460, USA

## Abstract

Understanding the mechanisms that drive acid-base regulation in organisms is important, especially for organisms in aquatic habitats that experience rapidly fluctuating pH conditions. Previous studies have shown that carbonic anhydrases (CAs), a family of zinc metalloenzymes, are responsible for acid-base regulation in many organisms. Through the use of phylogenetic tools, this present study attempts to elucidate the evolutionary history of the *α*-CA superfamily, with particular interest in the emerging model aquatic organism *Daphnia pulex*. We provide one of the most extensive phylogenies of the evolution of *α*-CAs, with the inclusion of 261 amino acid sequences across taxa ranging from Cnidarians to *Homo sapiens*. While the phylogeny supports most of our previous understanding on the relationship of how *α*-CAs have evolved, we find that, contrary to expectations, amino acid conservation with bacterial *α*-CAs supports the supposition that extracellular *α*-CAs are the ancestral state of animal *α*-CAs. Furthermore, we show that two cytosolic and one GPI-anchored *α*-CA in *Daphnia* genus have homologs in sister taxa that are possible candidate genes to study for acid-base regulation. In addition, we provide further support for previous findings of a high rate of gene duplication within *Daphnia* genus, as compared with other organisms.

## 1. Introduction

Organisms experience a variety of environmental stressors to which they must respond in order to survive and reproduce. Some are able to adjust to these stressors and live to produce offspring and propagate their genes, while others do not and are extirpated. There has been a plethora of work attempting to elucidate the changes in physiological and genetic mechanisms in response to human-induced stresses/impacts on aquatic habitats, including nutrient enrichment and cultural eutrophication [1–3], anthropogenically elevated carbon dioxide [4], and toxic metal contamination [5, 6]. Another important human-mediated impact to aquatic habitats, lake acidification/alkalization, has also been well studied [7–11].

Acidification and alkalization of water bodies are important ecological stressors that affect the structure of plankton communities. Although the processes of acidification and alkalization can occur naturally through mechanisms such as bedrock leaching [12], catchment runoff [10], and chemical conversion [13, 14], increasing impacts from anthropogenic sources such as carbon dioxide emissions [11], cultural eutrophication [7], and mining activities [6, 9] are of great concern with regard to lake acidification and alkalization.

**Figure 1:**
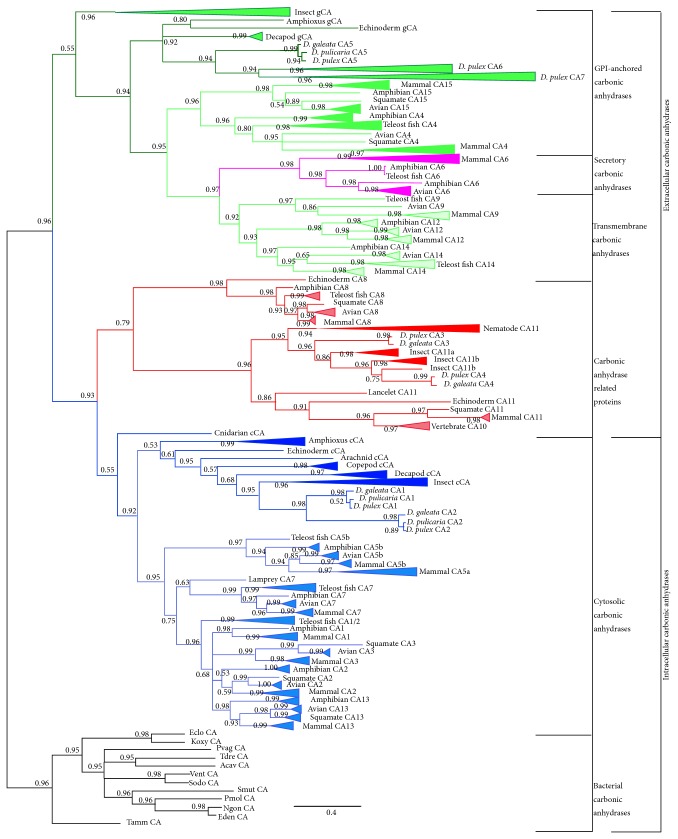
Phylogeny of *α*-CAs inferred from a maximum likelihood analysis performed with MrBayes; posterior probabilities of branches are indicated at the nodes. Species are collapsed within a larger taxonomical grouping. Branches are colored according to alpha-carbonic anhydrase families: GPI-anchored (dark green: invertebrate; medium green: vertebrate), membrane-bound (light green), secretory (purple), CA-RP (red), and cytosolic (dark blue: invertebrate; light blue: vertebrate). Black branches represent the bacterial outgroup *α*-CA families.

Maintaining pH homeostasis in these altered habitats is critical for organisms to survive and reproduce. Acid-base regulation in a number of aquatic organisms (e.g., fish [15–17], decapods [18, 19], and aquatic insects [20]) has been linked to the enzyme carbonic anhydrase (CA). CAs are zinc metalloenzymes that catalyze the reversible hydration/dehydration reaction: CO_2_ +  H_2_O  ⇔ H_2_CO_3_  ⇔  HCO_3_^−^ + H^+^, and are fundamental to many biological processes in addition to acid-base regulation, for example, photosynthesis [21], respiration [18, 22], osmoregulation [18, 22], bone resorption [23], and biominerization [24]. CAs are classified into five evolutionarily distinct and unrelated superfamilies: *α*, *β*, *γ*, *δ*, and *ζ*; each superfamily has different active site amino acids, primary sequences, and protein structure [25, 26]. These families are thought to be the result of convergent evolution. The *α*-CA superfamily typically has 16 or 17 different isoforms within vertebrates, which are the primary contributors to acid-base regulation. The *α*-CA superfamily is broken into four families: cytosolic, secretory, transmembrane/membrane-bound, and CA-related proteins (CA-RP), the latter of which have purportedly lost function due to the loss of at least one of the three active site Histidine residues [27]. In fish, decapods, and aquatic insects, the cytosolic and membrane-bound *α*-CAs in gills have been shown to regulate internal pH (Figure 1). The *β*-CAs are typically found only in bacteria, plants, algae, and fungi; however *β*-CAs have recently been found in some animals such as* Caenorhabditis elegans* [28],* Anopheles gambiae* [29],* and Daphnia pulex* [22]. There is a lack of knowledge on the catalytic activity and expression of *β*-CAs in animals, but in plants they are catalytically similar to *α*-CAs in animals. The *γ*-CAs have only been found in archaea and bacteria, while *δ*-CAs and *ζ*-CAs have only been found in marine diatoms [24]. The *ζ*-CAs are unique among CAs since they replace the zinc ion with cadmium [30].

In this study, we investigated the evolutionary history of *α*-CAs in the microcrustacean* Daphnia* genus using phylogenetic methods. Since little is understood about *β*-CAs in animals, this study focuses on *α*-CAs.* Daphnia* spp. are keystone aquatic herbivores and an emerging model organism, whose genome has been sequenced and annotated [27]. Interestingly, the* D. pulex* genome has a high rate of gene duplication, three times as high as* Drosophila* genus and nematodes and 30% higher than humans [27]. Since* Daphnia* spp. have 30 isoforms [22] of *α*-CAs, compared to the 15 in other organisms; this lends itself to the notion that there have been multiple duplication events within the* Daphnia α*-CAs. It has been hypothesized that duplication events can be a source for evolutionary novelties and that these duplications can follow one of several evolutionary trajectories: (i) one copy may become silenced (nonfunctionalization); (ii) one copy may acquire a novel beneficial function (neofunctionalization); or (iii) both copies may experience reduced functionality (subfunctionality) [31–33].

In addition, we used the phylogenetic analysis of the superfamily of *α*-CAs to clarify which* Daphnia α*-CAs may be investigated further for their role in acid-base regulation. The criteria for this analysis involved examining* Daphnia α*-CA genes with functioning *α*-CA homologs in other crustaceans [18, 19], aquatic insects [20], and fish [15–17]. Further, we investigated the evolutionary history of *α*-CAs in* Daphnia* to elucidate the functionality of duplicate *α*-CA genes, if they indeed exist.

## 2. Materials and Methods

### 2.1. Sequence Retrieval

All sequences, except* Daphnia* sequences, were obtained from the National Center for Biotechnology Information (NCBI). A key word search for “*Homo sapien* Carbonic Anhydrase” was performed for each of the 16 human isoforms of *α*-CA and the amino acid sequences were obtained. For each human isoform, a BLAST search was performed using the BLASTP algorithm with default settings from the National Center for Biotechnology Information (NCBI) (http://www.ncbi.nlm.nih.gov/blast/Blast.cgi). Only protein sequences from all taxa with an *E* value lower than e^−75^ were selected for analysis. The list of sequences was screened to ensure there were no duplicate sequences, based upon 100% sequence conservation in the gene within a given species. Partial sequences were discarded in the final analysis. Twelve *α*-CA amino acid sequences were retrieved from bacteria to use as an outgroup. This search resulted in 213 amino sequences from taxa ranging from cnidarians to mammals (Table S1 in Supplementary Material available online at http://dx.doi.org/10.1155/2015/538918). Our final list of taxa included more vertebrates than invertebrates; this bias is the result of the lack of whole genomes or CA loci sequences within invertebrates. While the bias towards more vertebrates does not affect the overall topology of the phylogeny, more invertebrates could have enhanced the resolution and support of the some of the invertebrate clades.

*Daphnia* sequences were obtained from the* Daphnia* Genomics Consortium (DGC) (http://wfleabase.org/). The* D. pulex* sequences were retrieved using the search function by entering the gene name. The nucleotide sequences were converted to amino acid sequences using MEGA 5.0 [34]. The* D. galeata* sequences were found by blasting the* D. pulex* CAs against the* D. galeata* database from the DGC using the TBLASTN algorithm with default settings [35].* D. pulicaria* were sequenced (Culver & Morton unpublished data) and converted to amino acid sequences using MEGA 5.0. This search resulted in 30* D. pulex*, 25* D. galeata*, and 3* D. pulicaria* amino acid sequences (Table S1). In addition, each* D. pulexα*-CA was mapped to their respective chromosome to infer duplication history and duplication events. We mapped each* D. pulexα*-CA isoform using known scaffold positions on their respective chromosomes [31].

### 2.2. Sequence Alignment and Phylogenetic Analysis

The 271 amino acid sequences were uploaded into CLUSTALX 2.1 [36] and a multisequence alignment was run with iterations after each alignment step. The aligned sequences were then uploaded into MEGA 5.0 and a best model fit was performed. The results from the best model fit indicated a Whelan and Goldman (WAG) model with gamma distribution and invariant sites [37]. Aligned amino acid sequences were then uploaded into the CIPRES web portal [38] and a Bayesian maximum likelihood phylogeny was created using MrBayes 3.1.2 [39] with the following parameters: 1 million iterations, 250 K burn-in, and 2 runs with 8 chains each. In addition, a bootstrapped maximum likelihood RaxML version 8.0 tree was constructed with 1000 iterations [40]. The resultant consensus tree was visualized using FigTree version 1.3.1 [41]; branches were collapsed for ease in reading the rather large phylogeny. Species composition of the collapsed branches can be found in Table S1. The aligned sequences were also used to determine residues that were conserved within each group of *α*-CAs (Table S2). A cutoff of 80% was used to determine if residues were conserved within an *α*-CA group across the entire phylogeny; however, if less than three species were in a group, then 100% conservation was used.

### 2.3. Determination of Ancestral States

Ancestral states of amino acids were inferred using a maximum likelihood approach within MEGA 5.0. Parameterization for the analysis employed a WAG model with gamma distribution and invariant sites and very strong branch swap filters. Criteria to elucidate the ancestral state of amino acids residues were determined by using those residues that are 80% conserved in the bacteria outgroup. This resulted in a reference sequence template that could be used to compare the other isoforms. Residues that were 90% conserved among all the isoforms in the phylogeny were excluded because they were not informative. Residues that were not shared among 50% of the isoforms in each *α*-CA group were also excluded to reduce noise. Twenty-seven residues remained for ancestral state analysis. As it is cumbersome to view the changes in ancestral states on the phylogeny, a table (Table S3) was created to facilitate a summary of amino acid residue evolution through the phylogeny. The table includes the predicted ancestral sequence at all nodes (the most recent common ancestor) and the number of amino acid changes from the most recent common ancestor (including homoplasies).

### 2.4. N-Terminus, GPI-Anchored, and Transmembrane Prediction of* Daphnia* CA6s and CA7s

To predict the transmembrane domains in the* Daphnia* CA6s and CA7s, the TMHMM Server v. 2.0 [42] on the Center for Biological Sequence Analysis (CBS) Prediction Server (http://www.cbs.dtu.dk/) was utilized. TMHMM uses a hidden Markov model to predict the location and likelihood of transmembrane helices. First, the amino acid sequences from the vertebrate extracellular CAs, determined from the phylogenetic analysis, were uploaded to the TMHMM server to determine if the software could successfully predict the known transmembrane CAs from the secretory and glycophosphatidylinositol- (GPI-) anchored *α*-CAs. The* Daphnia* CAs amino acid sequences were then uploaded into the TMHMM server. Those sequences that had a posterior probability greater than 0.80 and no N-terminus signal peptides were predicted to be transmembrane CAs.

To determine N-terminal sequences and cleavage sites in the* Daphnia* CA6s and CA7s, we used the TargetP 1.1 Server [43] on the CBS Prediction Server. As with predicting transmembrane domains, amino acid sequences from known vertebrate extracellular CAs, as determined by the phylogenetic analysis, were uploaded into the TargetP 1.1 Server with the following parameters: (i) nonplant organisms; (ii) cleavage sites predicted; and (iii) a specificity cutoff of greater than 0.7. This run was used to determine if the software could successfully predict the *α*-CAs with known N-terminus sequences.* Daphnia* CAs amino acids from the CA6s and CA7s were then uploaded in the TargetP 1.1 Server with the same parameters to predict N-terminus signal peptides.

GPI-anchored proteins in* Daphnia* genus were predicted using the online based software, GPI-SOM (http://gpi.unibe.ch/) [44]. GPI-SOM uses a Kohonen self-organizing mapping approach to predict C-terminus anchoring signal and anchoring site. GPI-anchoring sites are only found in the C-terminus of a protein. GPI-anchoring proteins also contain N-terminus signaling peptides. Known vertebrate extracellular amino acid sequences, as determined from the phylogenetic analysis, were uploaded into GPI-SOM and were run with default parameters. The results were used to elucidate whether GPI-SOM could successfully predict the known GPI-anchored *α*-CAs from the secretory and transmembrane *α*-CAs. Amino acids from* Daphnia* CA6s and CA7s were uploaded into GPI-SOM to predict* Daphnia* GPI-anchored *α*-CAs.

The prediction software was able to successfully place the vertebrate *α*-CA subfamilies into their respective subfamilies, for instance transmembrane, secretory, and GPI-anchored *α*-CAs.

## 3. Results and Discussion

### 3.1. General Phylogenetic Distribution of CA Types in Animals

Typically *α*-CAs are characterized by (i) four active site residues: Histidine- (His-) 316 (His-64 using nomenclature of vertebrate *α*-CAs), Glutamine- (Gln-) 353, Glutamic Acid- (Glu-) 372, and Threonine- (Thr-) 498; (ii) three zinc-binding site residues: His-355, His-357, and His-385; and (iii) two substrate-binding site residues: Thr-498 and Thr-499. The residue His-316 acts as a proton shuttle from the zinc ion and is considered a rate-limiting step in the catalytic process [45]. So the inclusion of the His at 316 is important in determining the activity level of the enzyme. Further, the residues Thr-498 and Thr-499 result in a threonine loop, which coordinates the zinc ion and is important in the overall activity of the enzyme [46]. The amino acid alignment shows that these residues are highly conserved throughout the phylogeny (Table S2). Also the residues surrounding these highly conserved residues have recognizable motifs that are also highly conserved. There are also three motifs that are highly conserved that are not associated sequentially with any of the active, zinc-binding, or substrate-binding sites: the motif QSPINI found at residues 219–224, GLAVLG found at residues 408–413, and N-RP-QPL at residues 570–577.

The phylogenetic results of the MrBayes (Figure 1) and RAxML (Figure S1) analyses produced similar topologies. The phylogenies indicate that the first divergence in *α*-CAs resulted in two sister clades representing extracellular and intracellular *α*-CAs and appeared after the split of animals, plants, and fungi from bacteria. Before this early divergence, the most likely ancestral state of the *α*-CAs was extracellular (which include the GPI-anchored, transmembrane/membrane-bound, and secretory *α*-CAs), as is evident by the bacterial *α*-CAs having similarly conserved residues as the extracellular *α*-CAs in animals (Table S3). Another line of evidence suggests that bacterial *α*-CAs are formed near to or on the cytoplasmic membrane [47]. In addition, Le Roy et al. [24] found that Porifera *α*-CAs were more similar to extracellular *α*-CAs and were more basal phylogenetically than intracellular *α*-CAs. In particular, both bacterial *α*-CAs and extracellular animal *α*-CAs share the same active site residues, zinc-binding site residues, and substrate-binding site residues. In addition, they have disulfide bonding sites at residues Cysteine- (Cys-) 214 and Cys-502 that are not found in cytosolic *α*-CAs (Tables S2 and S3). Further, extracellular *α*-CAs and bacterial *α*-CAs share the following conserved residues that are not found in intracellular *α*-CAs (however, they are found in some of the CA-RPs): Asparagine- (Asn-) 314, Asn-434, Tyrosine- (Tyr-) 491, Arginine- (Arg-) 492, and Arg-578 (Table S3). These results are contrary to the commonly held notion that the intracellular *α*-CAs are the ancestral state [25].

The GPI-anchored *α*-CAs, found within the extracellular *α*-CAs, form three monophyletic clades: these clades consist of an invertebrate clade (including the chordate amphioxus), vertebrate clade, and an insect clade. Of note is the fact that insects did not fall within the invertebrate clade; however, the insect GPI-anchored clade has weak support (posterior probability = 0.55, Figure 1). The vertebrate subclade of GPI-anchored *α*-CAs is characterized by CA4 and CA15. In vertebrates, CA4 is localized in the kidneys, gastrointestinal tract, and endothelium, while CA15 is localized in the kidneys and is not expressed in humans [45, 47]. One of the weaknesses of constructing robust phylogenies of metazoans *α*-CAs is that there is a lack of depth of taxon sequence coverage in invertebrate organisms. Some researchers choose to limit their analysis to organisms that have whole genome sequences, in order to increase the likelihood of capturing all isoforms; however, this limits the number of taxa that can be used. We chose to use both whole genomes and individually sequenced *α*-CA isoforms to increase coverage of both isoforms and taxa [24]. However, even taking this approach, there is a severe lack of data on sequenced *α*-CAs within invertebrates, thus weakening support for some relationships within the phylogeny. This may also cause some sampling bias when trying to deduce the rate of duplication events between invertebrate taxa. With the plethora of next-generation studies taking place, perhaps this lack in data will be resolved in the near future.

The GPI-anchored *α*-CAs further diverged into the secretory type *α*-CAs due to the loss of the C-terminus cleavage and anchoring site [22, 46], which occurred after the appearance of amphioxus. This can be deduced, since the secretory *α*-CA appears in all vertebrates. The secretory *α*-CA is characterized by CA6s, which is localized in the saliva of vertebrates. Membrane-bound *α*-CAs diverged from a common ancestor with the secretory *α*-CAs based on phylogenetic support that shows the divergence occurring after amphioxus, but before the amphibian/fish divergence. The transmembrane *α*-CAs are characterized by the further loss of the N-terminus signal peptides and the development of helices that are embedded in the cell membrane and are represented by CA9, CA12, and CA14.

After the split of animal phyla, extracellular *α*-CAs diverged from intracellular *α*-CAs. The intracellular *α*-CAs are characterized by an amino acid change from the ancestral state at the following residues: 233 from Isoleucine (Ile) to Proline (Pro), 314 from Asn to Thr, 318 from Ile to Serine (Ser), 319 from Gln to Phenylalanine (Phe), 448 from Ile to Thr, 491 from Tyr to Trytophan (Trp), 492 from Arg to Thr, and 505 from Glysine (Gly) to Ser (Table S3). During the evolution of intracellular *α*-CAs, a duplication event likely occurred, which split intracellular *α*-CAs into two clades: CA-related proteins (CA-RPs) and cytosolic *α*-CAs. The CA-RPs are characterized by an amino acid change at the active site residue 353 from Gln to Glu in all the CA-RPs, an amino acid change at the active site residue 316 from His to Ser in the CA11s, and an additional amino acid change at the zinc-binding site residue 385 from His to Gln in the CA10s and CA11s, which resulted in the complete loss of function (nonfunctionalization) or a different function (neofunctionalization) in these enzymes [45]. According to the phylogeny, this duplication must have occurred before the emergence of cnidarians.

The CA-RPs form a large monophyletic group made up of three subclades. One subclade consists of CA8 and contains only deuterostomes. An interesting feature of the CA8 subclade is that there are relatively short branch lengths across a diverse group of taxa, suggesting high conservation within this subclade despite these isoforms being noncatalytic. These results suggest that CA8 may have an important biological function within deuterostomes [27]. Further, the CA8 subclade is sister to both CA11s and CA10s. The CA11s (including* Daphnia* CA3 and CA4, nomenclature for the *α*-CAs of* Daphnia* genus and many invertebrates are not consistent with the nomenclature of *α*-CAs used for mammals) form two distinct groupings: protostomes and deuterostomes.

The cytosolic *α*-CAs make up a monophyletic group and are characterized by the loss of the disulfide bond at residue 214 due to the Cys converting to different amino acids that do not facilitate disulfide bonds. The loss of the disulfide bond at residue 214 in the cytosolic *α*-CAs suggests relaxed selection, since these enzymes do not need the extra structural integrity provided by the disulfide bond to deal with the environment outside the cell [24, 46]. Also of interest within the cytosolic *α*-CAs is the fact that there was an amino acid change at the active site residue 316 from His to Asn in vertebrates and to a Thr in invertebrates, with the subsequent reemergence of the His at residue 316 in vertebrate CA1, 2, 7, and 13, and fish CA1/2 (Table S3). The His-316 residue is important in the activity of the enzyme in that it acts as a proton shuttle from the zinc ion and is considered a rate-limiting reaction [46]. In addition, vertebrate CA1 and CA13 have a conversion of Thr-499, an important residue in the coordination of the zinc ion and is important in catalytic activity, to His in CA1 and Valine (Val) in CA7. This supports why CA2 has been determined to be the most active of the vertebrate cytosolic *α*-CAs, while the others have varying degrees of lower activity [46]. Within the cytosolic *α*-CAs, after the (weakly supported: posterior probability = 0.55) divergence of cnidarians, there is a split resulting in an exclusively vertebrate clade and a clade containing all the invertebrates (including amphioxus). The most basal group of the vertebrate clade consists exclusively of CA5, which is associated with mitochondria [46], with the next divergence from the CA5/7 common ancestor being CA7 followed by the teleost fish CA1/2. The teleost fish CA1/2 clade shows evidence of a duplication event [48, 49]; however, it is not universal to all teleosts. This is represented within the collapsed clade of teleost fish CA1/2 of the cytosolic *α*-CAs (Figure 1). In addition, after the divergence of teleost fish CA1/2, a polytomy is formed and the relationship among CA1, CA2, CA3, and CA13 type cytosolic *α*-CAs cannot be resolved. Here, the RAxML tree resolves the polytomy but has relatively weak support (bootstrap values = 19–44, Figure S1). The sister group to the exclusively vertebrate subclade contains all the invertebrates and amphioxus.

### 3.2. *Daphnia* CA Isoforms

Of the* Daphnia α*-CAs, two fall within the cytosolic family (CA1 and CA2), clustering with other arthropods, echinoderms, and cnidarians (Figure 1).* Daphnia* CA5 clusters with GPI-anchoring *α*-CAs of other arthropods (Figure 1). Two other *α*-CAs (CA3 and CA4) cluster within the CA-RP clade and are sisters to hexapod CA-RPs (Figure 1). Specifically, CA3 is closely associated with hexapod CA11a, while CA4 is sister to hexapod CA11b.

**Figure 2:**
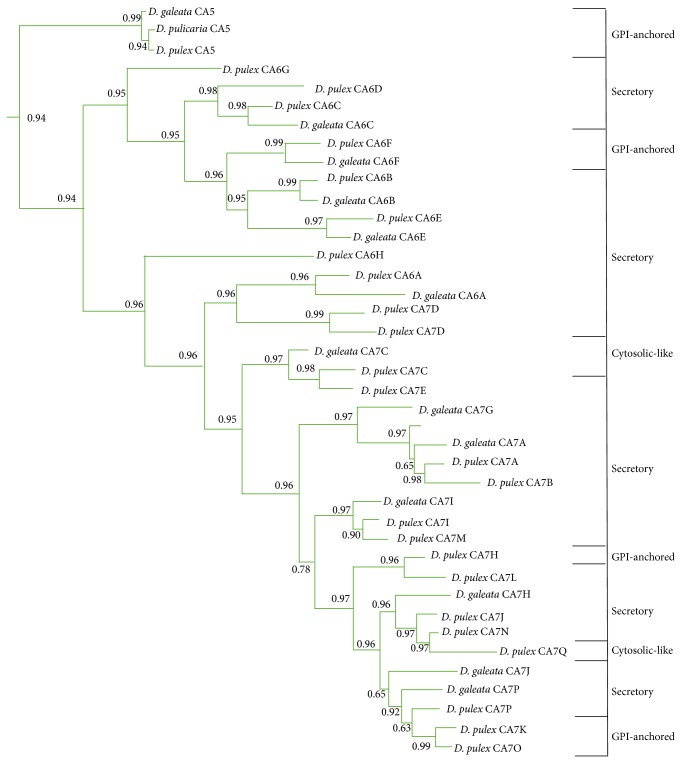
Isolated view of* Daphnia* CA5, CA6s, and CA7s based on the phylogeny represented in Figure 1. Posterior probabilities of the branches are indicated at the nodes. On the right side of the phylogeny are the predicted states for* Daphnia* CA5, CA6s, and CA7s.

The remaining 25 *α*-CAs form two sister clades, CA 6B-G and CA7A-Q (including CA6A and CA6H), that diverged from CA5. In previous work [22], CA6H was the first branch in the CA6s clade, while CA6A was excluded from the phylogeny. Since in this study CA6A and CA6H cluster with the CA7s, we would propose to rename these genes as CA7R and CA7S, respectively, since each of the nodes have good posterior probability support (0.96) (Figure 2). Weber and Pirow [22] suggest that CA6s and CA7s are secretory CAs due to fact that they have N-terminus signaling peptides; however, our analysis does not support that all the CA6s and CA7s are secretory. Using transmembrane, N-terminus, and GPI-anchoring software we found that, like Weber and Pirow, none of the CA6s or CA7s are transmembrane *α*-CAs using a posterior probability cutoff of >0.8. We did find evidence to support that* Daphnia* CA6F, CA7H, CA7K, and CA7O are GPI-anchored *α*-CAs in that they all had N-terminus signaling peptides and C-terminus cleavage and anchoring sites (Table 1 and Figure 2). All the remaining* Daphnia* CA6s and CA7s, except CA6E and CA7Q, had N-terminus signaling peptides (specificity > 0.7) without the C-terminus cleavage and anchoring sites, suggesting that these *α*-CAs are secretory (Table 2, Figure 2). The two remaining* Daphnia α*-CAs, CA6E and CA7C, were not predicted to be transmembrane, secretory, or GPI-anchoring proteins and may have some cytosolic function (Table 1, Figure 2). Le Roy et al. [24] also found cytosolic-like CAs in poriferans and mollusks that are involved in biocalcification in *α*-CAs within their respective extracellular clade. They suggest that this may be an internalization of a formally secreted *α*-CA or they may be secreted proteins that are shuttled out of the cell in a novel manner. Further research is warranted to verify the function and localization of these *α*-CAs.

### 3.3. Duplication Events in CA Isoforms in* Daphnia* Genus

Phylogenetic results also support the hypothesis of multiple duplication events in* Daphnia* genus. The first duplication event seems to be the result of gene-level duplication in an ancestral species that resulted in the divergence of cytosolic *α*-CAs from extracellular *α*-CAs and CA-RPs. This is supported by the fact that extracellular *α*-CAs and CA-RPs (the predicted ancestral state of *α*-CAs), as a group, are found in tandem on chromosome 7 (Table 2). The second duplication is the result of a genome-level duplication event in an ancestral species, which led to the divergence of the CA-RPs from the cytosolic *α*-CAs. Evidence in support of this is that the CA-RPs (and extracellular *α*-CAs) are found on chromosome 7, while the cytosolic *α*-CAs are found on chromosome 4. Since,* Daphnia *spp. are known to have a high level transposable elements [27], this could be a potential mechanism through which the gene was able to move within the genome. Another potential mechanism could be chromosome duplication. Further investigation is needed to determine which mechanism is supported. As with the already discussed isoforms, the remaining isoforms (CA1 and CA2, CA3 and CA4, and CA6s and CA7s) also appear to be the result of duplication events. One possibility is that these duplications are the result of tandem duplications, as many of the genes are in synteny (Table 2). Although there is only the one GPI-anchored *α*-CA in* Daphnia* genus (CA5), there is a radiation of 25 *α*-CAs (CA6A-H and CA7A-Q), which diverged from CA5. When the CA6s and CA7s diverged from CA5, they lost their GPI-anchoring site but retained the N-terminus signaling peptide sequence allowing for neofunctionalization as secretory *α*-CAs (Table 1 and Figure 2). Four isoforms, CA6F, CA7H, CA7K, and CA7O, later reverted to GPI-anchored *α*-CAs through convergent evolution. Additionally, two isoforms, CA6E and CA7Q, lost both the N-terminus signaling peptide and C-terminus cleavage sequence. This suggests that they either became cytosolic or developed a novel secretory pathway [24]. Several studies of* Daphnia* genus and other invertebrate genes and genomes have unveiled duplicated genes that have led to neofunctionalization, such as the spooky genes in arthropods [50]. If these duplications prove to be neofunctional, then* Daphnia α*-CAs would have a larger than expected number of neofunctional isoforms. Kondrashov [51], in his review, explains how it is possible for duplicated genes to persist in the genome long enough to eventually evolve into neofunctional genes through the redundancy hypothesis, which postulates that duplicate genes are not deleterious but are maintained through neutral processes and can evolve into neofunctional genes if they lead to a fitness advantage.* Daphnia's* two CA-RPs do not appear to be duplicated within the genus but belong to a larger duplication within the phylum Arthropoda that occurred after the divergence of arthropods and nematodes.

The fact that* Daphnia* genus has 30 isoforms of *α*-CA, while most vertebrates only have 15 or 16, lends support to previous work on the* Daphnia* genome, which found that* Daphnia* genus has a relatively high rate of gene duplication, at least within* D. pulex*. These high duplication rates are not novel to* Daphnia* genus and have been shown in another cyclically parthenogenetic organism, the pea aphid,* Acyrthosiphon pisum* [52, 53].

The phylogeny presented here shows that this may be a genus-wide phenomenon, as* D. galeata* also shares this radiation event within the CA6s and CA7s; however, the* D. galeata* radiation is not as extensive: 20 isoforms of CA6s and CA7s, as compared to 25 in* D. pulex*. As the genomes of two additional daphniid species (i.e.,* D. magna* and* D. pulicaria*) are completed, it will be of interest to determine if these genomes support the finding of a large radiation of CA6s and CA7s within the genus* Daphnia*. It also appears that CA1 and CA2 are the products of duplication within the genus, whereas the duplication of CA3 and CA4 appears to be within the whole arthropod phylum; however, as more arthropod genomes are sequenced, this may fill in gaps in the phylogeny.

## 4. Conclusions

The results of this phylogenetic study support the previously held organization of the *α*-CA superfamily of genes, namely, the fact that *α*-CAs are clustered into the following families: cytosolic, CA-RP, GPI-anchored, secretory, and membrane-bound [25]. Previous thought, however, was that intracellular *α*-CAs were the most likely ancestral state. In contrast, our results provide support that extracellular *α*-CAs are the likely ancestral state. The added knowledge from this extensive phylogeny elucidates the relationship among invertebrates and vertebrates. For instance, the GPI-anchored and cytosolic *α*-CAs are divided into invertebrate and vertebrate groups. The nomenclature that is used for the vertebrate CAs does not hold up when looking at invertebrate groups. For instance, some of the cytosolic invertebrate CAs are named CA1, CA2, or cCA but do not have any phylogenetic relationship to vertebrate CA1 or CA2. In fact the invertebrate *α*-CAs are more closely related to the more basal vertebrate *α*-CAs, CA7 and CA5 (Table S3). Since the vertebrate CA5 is associated with mitochondria, invertebrate cytosolic *α*-CAs are, therefore, more similar to vertebrate CA7. This is also true for the extracellular *α*-CAs; however, since there are many homoplasies occurring within each invertebrate taxonomical clade, it is difficult to determine their relationship to the established nomenclature of vertebrate extracellular *α*-CAs. Further, invertebrate groups have reduced diversity of cytosolic and extracellular *α*-CAs, when compared to vertebrates. However, this may be an artifact of the fact that invertebrate *α*-CAs have not been well investigated. A study of the purple sea urchin (*Strongylocentrotus purpuratus*) has uncovered 19 isoforms of *α*-CA, most of which are involved in acid-base regulation [54]. Also,* D. pulex* has 30 different isoforms [22]. Most of these isoforms are the result of a radiation of CA6s and CA7s that diverged from* Daphnia* CA5, which is a GPI-anchored *α*-CA. Further investigation of invertebrate *α*-CAs may uncover a greater diversity of *α*-CAs within these families.

We had several overarching goals in performing this study with regard to the* Daphnia* genus. First, we were interested in the evolution of* Daphnia α*-CAs. Homologs of acid-base regulating *α*-CAs in organisms, such as crustaceans, aquatic insects, and fish were used to provide evidence in support of potential acid-base regulating *α*-CAs in* Daphnia* genus. Second, we were interested in gene duplication events in the *α*-CA superfamily, specifically within the* Daphnia* genus, and the fate of these duplicated genes evolutionarily.

To address the goal of identifying potential genes involved in acid-base regulation in* Daphnia* genus, several candidate genes, including CA1, CA2, and CA5, may be implicated as a starting point for investigation. Since these three genes have homologs in other arthropods that have been previously determined physiologically to be active in acid-base regulation [18], these* Daphnia* CA genes warrant further study (e.g., physiological fitness assays across a range of pH conditions) to determine their functionality. To date, it is uncertain what the expression levels of *α*-CAs are in* Daphnia* genus. In other organisms, however, some experiments have shown differential expression of *α*-CAs across a pH gradient. For instance, Evans et al. [54] found that *α*-CA12 was differentially expressed in larval* S. purpuratus* at low pH conditions. Also, Lin et al. [16] found differential expression of *α*-CA2 and CA15 in the gills of zebrafish under differing pH conditions. Current work in our lab is trying to characterize these genes and elucidate the differential expression of *α*-CAs across pH gradients. Furthermore, this study can be useful as a reference for any future acid-base regulation work in other arthropods, particularly crustaceans.

Within* Daphnia* CAs, there is a major radiation within the CA6 and CA7s of 25 CA isoforms, which diverge from CA5. In addition, CA1/CA2 and CA3/CA4 represent additional, independent duplications compared to other arthropods. This recurrent observation of multiple duplication events in* Daphnia* genus lends support to the hypothesis that the ecoresponsive nature of this organism may be due to possible neofunctionaliztion resulting from the high levels of gene duplication. Thus, the genome duplications in* Daphnia* genus may allow this organism to withstand an extensive range of environmental (e.g., pH) conditions that are encountered in aquatic habitat [27].

## Supplementary Material

The supplementary material that follows includes the following tables and figures: Tables S1, S2, S3, and Figure S1.
We constructed Table S1 to list the taxa with their respective isoforms of α-Carbonic Anhydrase (CA) that were used in both the MrBayes and RaxML phylogenetic analysis. Since, branches of the resultant phylogenies are collapsed for clarification of the figures, it was necessary to provide a list of the taxa used. We also provide the NCBI ascension number of the CAs used.
Table S2 is listing of the conserved amino acid residues among the α-CA isoforms and taxonomical groupings. It was compiled using the alignment of the amino acid sequences (see Material and Methods). Residues that are conserved within α-CA groupings are highlighted by a color based upon which grouping they are associated (see Table legend).
Table S3 list the ancestral states of informative amino acid residues (see Materials and Methods for criteria) based upon the phylogeny. Each node of the phylogeny is represented in the table with the predicted ancestral amino acid sequence. The ancestral states were inferred using a maximum likelihood approach.
Figure S1 is the result of our RaxML phylogenetic analysis. This tree has a similar topology as the MrBayes tree that is printed in the main manuscript. This tree was run to verify or MrBayes results. The main difference between these two trees is that the RaxML tree resolves the polytomy in the vertebrate cytosolic α-CAs, but with low support.

## Figures and Tables

**Table 1:** Results of prediction software to determine whether proteins are transmembrane, secretory, or GPI-anchored. Transmembrane proteins were determined using the TMHMM server on the CBS Prediction Server with a posterior probability >0.8 and no N-terminus prediction on a transmembrane protein. N-terminus signaling peptides were elucidated using TargetP on the CBS Prediction Server, with a specificity >0.7 indicating a high probability of a N-terminus signaling peptide. GPI-SOM was used to predict C-terminus cleavage and anchoring sites. If a protein was not transmembrane and has both N-terminus and C-terminus it was predicted to be a GPI-anchored protein. If it had only a N-terminus prediction, it was classified as secretory protein. If it did not fit any category it was classified as a cytosolic-like protein.

*Daphnia* CA isoform criteria ->	Transmembrane >0.8 and no N-terminus	N-Terminus >0.7 specificity	C-Terminus Most probable	Prediction
CA5	No	Yes	Yes	GPI-anchored
CA6A	No	Yes	Not	Secretory
CA6B	No	Yes	Not	Secretory
CA6C	No	Yes	Not	Secretory
CA6D	No	Yes	Not	Secretory
CA6E	No	0.178	Not	Cytosolic-like
CA6F	No	Yes	Yes	GPI-anchored
CA6G	No	Yes	Not	Secretory
CA6H	No	Yes	Not	Secretory
CA7A	No	Yes	Not	Secretory
CA7B	No	Yes	Not	Secretory
CA7C	No	Yes	Not	Secretory
CA7D	No	Yes	Not	Secretory
CA7E	No	Yes	Not	Secretory
CA7F	No	Yes	Not	Secretory
CA7G	No	Yes	Not	Secretory
CA7H	No	Yes	Yes	GPI-anchored
CA7I	No	Yes	Not	Secretory
CA7J	No	Yes	Not	Secretory
CA7K	No	Yes	Yes	GPI-anchored
CA7L	No	Yes	Not	Secretory
CA7M	No	Yes	Not	Secretory
CA7N	No	Yes	Not	Secretory
CA7O	No	Yes	Yes	GPI-anchored
CA7P	No	Yes	Not	Secretory
CA7Q	No	0.288	Not	Cytosolic-like

**Table 2:** Results of chromosome mapping which reflect the *D. pulex α*-CA isoforms and their scaffold designation along with their start and end positions on the scaffold. Three isoforms could not be mapped to a chromosome because their scaffolds have not been mapped to their respective chromosome.

*D. pulex* CA	Scaffold	Start position	End position	Chromosome	Dappu ID
CA1	8	293280	297489	4	442498
CA2	8	1005314	1007373	4	442497
CA3	74	63490	73363	NA	442499
CA4	4	1033301	1039412	7	442496
CA5	20	1028754	1037862	NA	442477
CA6A	4	1676667	1677698	7	442779
CA6B	4	1678702	1680800	7	442471
CA6C	4	1682181	1683985	7	442472
CA6D	4	1687613	1689716	7	442467
CA6E	4	1692426	1694512	7	442475
CA6F	4	1699762	1703139	7	442468
CA6G	4	1707093	1708695	7	442476
CA6H	4	2922515	2924220	7	442478
CA7A	4	2427959	2429626	7	442480
CA7B	4	2430816	2432334	7	442481
CA7C	4	2435092	2435571	7	442482
CA7D	4	2436638	2438161	7	442483
CA7E	4	2438986	2440394	7	442484
CA7G	4	1707093	1708695	7	442494
CA7H	4	2463490	2465025	7	442485
CA7I	4	2466064	2467469	7	442486
CA7J	4	2468464	2470065	7	442487
CA7K	4	2470727	2472139	7	442488
CA7L	4	2474751	2475358	7	442489
CA7M	4	2477904	2479557	7	442491
CA7N	4	2480236	2482046	7	442490
CA7O	4	2482392	2383774	7	442492
CA7P	4	2486891	24888402	7	442493
CA7Q	40	788747	790739	NA	442495
