# Supplementary material for: The Evolutionary History of Daphniid α-Carbonic Anhydrase within Animalia
Source: Int J Evol Biol. 2015 Mar 29;2015:538918. doi: 10.1155/2015/538918 (PMC4393933; doi:10.1155/2015/538918)

Table S1 List of the α-CA sub-families with the species and accession number of the amino acid sequences used in this study.

α-CA Sub-Family	Species Name	Accession #
CA 10	Homo sapiens_CA10	AAH47456.1
	Mus musculus_CA10	AHH17606.1
	Macaca mulatta_CA10	AFE64410.1
	Equus caballus_CA10	XP_001503286.2
	Bos taurus_CA10	AAI26508.1
	Gallus gallus_CA10	XP_415644.1
	Taeniopygia guttata_CA10	XP_002198310.1
	Anolis carolinesis_CA10	XP_003216966.1
	Rattus norvegicus_CA10	XP_002727818.1
	Xenopus tropicalis_CA10	XP_002932546.1
	Danio rerio_CA10	NP_001032198.1
	Oreochromis niloticus_CA10	XP_003438532.1
CA11	Macaca mulatta_CA11	AFE64868.1
	Bos taurus_CA11	AAI23828.1
	Homo sapiens_CA11	AAH02662.1
	Mus musculus_CA11	AAH19392.1
	Rattus norvegicus_CA11	AAL78173.1
	Loxodonta africana_CA11	XP_003406877.1
	Anolis carolinesis_CA11	XP_003225067.1
	Stronglocentrotus purpuratus_CA11	XP_784796.2
	Branchiostoma floridae_CA11	XP_002611575
	Drosophila melanogaster_CA11a	NP_572581.1
	Tribolium castaneum_CA11a	XP_972474.2
	Nasonia vitripennis_CA11a	XP_003424234.1
	Drosophila melanogaster_CA11b	NP_572407.3
	Aedes aegyti_CA11	XP_0016588761.1
	Tribolium castaneum_CA11b	XP_974173.2
	Daphnia pulex_CA4	EFX88377.1
	Daphnia galeata_CA4	unpublished
	Daphnia pulex_CA3	EFX72738.1
	Daphnia galeata_CA3	unpublished
	Loa loa_CA11	XP_003137734.1
	Brugia malayi_CA11	XP_001900835.1
	Caernorhabditis elegans_CA11a	NP_495567.3
	Trichinella spiralis_CA11	XP_003376534.1
	Caernorhabditis elegans_CA11b	NP_498083.1
CA 8	Homo sapiens_CA8	EAW86826.1
	Macaca mulatta_CA8	EHH28528.1
	Mus musculus_CA8	AAH10773.1
	Rattus norvegicus_CA8	AAH87586.1
	Bos taurus_CA8	NP_001077159.1
	Equus caballus_CA8	XP_001496523.1
	Gallus gallus_CA8	XP_419221.3
	Meleagris gallopavo_CA8	XP_003205121.1
	Taeniopygia guttata_CA8	XP_002197204>1
	Anolis carolinesis_CA8	XP_003219630.1
	Xenopus tropicalis_CA8	NP_001011213.1
	Danio rerio_CA8	AAH92740.1
	Oncorhynchus mykiss_CA8	NP_001118116.1
	Stronglocentrotus purpuratus_CA8	XP_795365.3
CA 2	Homo sapiens_CA2	NP_000058.1
	Macaca mulatta_CA2	NP_001182346.1
	Equus caballus_CA2	XP_001488540.1
	Bos taurus_CA2	NP_848667.1
	Mus musculus_CA2	EDL05130.1
	Rattus norvegicus_CA2	NP_062164.1
	Gallus gallus_CA2	NP_990648.1
	Taeniopygia guttata_CA2	XP_002199833.1
	Anolis carolinesis_CA2	XP_003219585.1
	Xenopus tropicalis_CA2	AAH89661.1
	Xenopus laevis_CA2	NP_001080080.1

Piscean CA1/2	Oncorhynchus mykiss_CA1	NP_001117692.1
	Dicentrarchus labrax_CA1	CBN82139.1
	Oncorhynchus mykiss_CA2	NP_001117693.1
	Ictalurus punctatus_CA2	NP_001187560.1
	Danio rerio_CA2	NP_954685.1
CA 1	Homo sapiens_CA1	NP_001729.1
	Macaca mulatta_CA1	EHH28617.1
	Equus caballus_CA1	XP_001488605
	Mus musculus_CA1	AAA37354.1
	Rattus norvegicus_CA1	AAI58889.1
	Bos taurus_CA1	AAI16127.1
	Xenopus tropicalis_CA1	XP_002939198
CA 13	Homo sapiens_CA13	AAH52602.1
	Macaca mulatta_CA13	XP_001095487.1
	Equus caballus_CA13	XP_001489984.2
	Mus musculus_CA13	AAK16672.1
	Rattus norvegicus_CA13	NP_001128465.1
	Bos taurus_CA13	DAA22563.1
	Gallus gallus_CA13	XP_003640859.1
	Meleagris gallopavo_CA13	XP_003205200.1
	Python molurus_CA13	AEA49961.1
	Anolis carolinesis_CA13	XP_003219587.1
	Xenopus tropicalis_CA13	NP_001072448.1
	Xenopus laevis_CA13	NP_001086981.1
CA 7	Petromyzon marinus_CA2	
	Homo sapiens_CA7	NP_005173.1
	Macaca mulatta_CA7	XP_001085299.2
	Mus musculus_CA7	NP_444300.1
	Rattus norvegicus_CA7	NM_001106165.1
	Loxodonta africana_CA7	XM_003417135.1
	Bos taurus_CA7	NP_001179451.1
	Gallus gallus_CA7	XP_41415.2
	Meleagris gallopavo_CA7	XP_003209867.1
	Taeniopygia guttata_CA7	XP_002190292.1
	Xenopus tropicalis_CA7	NP_001015903.1
	Danio rerio_CA7	NM_200813.1
	Ictalurus punctatus_CA7	NP_001187680.1
	Oreochromis niloticus_CA7	XM_003445759.1
CA 5	Homo sapiens_CA5a	NP_001730.1
	Macaca mulatta_CA5a	EHH31930.1
	Equus caballus_CA5a	XP_001500362.2
	Bos taurus_CA5a	DAA20167.1
	Mus musculus_CA5a	AAH30174.1
	Rattus norvegicus_CA5a	AAH88147.1
	Homo sapiens_CA5b	NP_00915.1
	Macaca mulatta_CA5b	EHH30558.1
	Equus caballus_CA5b	XP_001490399.1
	Bos taurus_CA5b	NP_001074377.2
	Mus musculus_CA5b	CAM26357.1
	Rattus norvegicus_CA5b	AAH81872.1
	Gallus gallus_CA5b	XP_414195.3
	Meleagris gallopavo_CA5b	XP_003209935.1
	Taeniopygia guttata_CA5b	XP_002192230.2
	Xenopus tropicalis_CA5b	CAJ82585.1
	Xenopus laevis_CA5b	NP_001084659.1
	Danio rerio_CA5b	NP_001104671.1
CA 3	Homo sapiens_CA3	3UYN
	Macaca mulatta_CA3	EHH28618.1
	Mus musculus_CA3	AAH11129.1
	Rattus norvegicus_CA3	AAH61980.1
	Equus caballus_CA3	NP_001157426.1
	Bos taurus_CA3	DAA22526.1

	Gallus gallus_CA3	NP_001264340.1
	Meleagris gallopavo_CA3	XP_003205196.1
	Taeniopygia guttata_CA3	XP_002199827.1
	Anolis carolinesis_CA3	XP_003219614.1
Invertebrate Cytosolic CA	Branchiostoma floridae_cCA2	XP_00259304.1
	Branchiostoma floridae_cCA3	XP_002605865.1
	Ixodes scapularis_cCA	XP_002416504.1
	Anthopleura elegantissima_cCA	AAD32675.1
	Stronglocentrotus purpuratus_cCA	XP_782997.2
	Drosophila melanogaster_cCA	AAF53332.1
	Anopheles gambiae_cCA	ABF66618.1
	Danaus plexippus_cCA	EHJ77985.1
	Tribolium castaneum_cCA	XP_974322.1
	Daphnia pulicaria_CA1	unpublished
	Daphnia pulex_CA1	EFX86103.1
	Daphnia galeata_CA1	unpublished
	Daphnia pulicaria_CA2	unpublished
	Daphnia pulex_CA2	EFX86394.1
	Daphnia galeata_CA2	unpublished
	Panaeus monodon_cCA	ABV65904.1
	Litopenaeus vannamei_cCA	ADM16544.2
	Callinectes sapidus_cCA	ABN51213.1
	Portunus trituberculatus_cCA	AFV46144.1
	Carcinus maenus_cCA	ABX71208.1
	Caligus rogercresseyi_cCA	ACO11717.1
	Caligus clemensi_cCA	ACO15216.1
	Lepeophtheirus salmonis_cCA	ACO12707.1
CA 4	Homo sapiens_CA4	NP_000708.1
	Macaca mulatta_CA4	XP_001107970.2
	Bos taurus_CA4	AAB09466.1
	Equus caballus_CA4	XP_001501182.1
	Mus musculus_CA4	AAH12704.1
	Rattus norvegicus_CA4	AAH97329.1
	Gallus gallus_CA4	XP_415893.1
	Python molurus_CA4	AEA49062.1
	Danio rerio_CA4	AAI09406
	Oreochromis niloticus_CA4	XP_003456174.1
	Xenopus tropicalis_CA4	XP_002983882.1
	Xenopus laevis_CA4	NP_001079788.1
CA 15	mus musculus_CA15	NP_085035.1
	Rattus norvegicus_CA15	NP_001099371.1
	Equus caballus_CA15	XP_005613908.1
	Gallus gallus_CA15	XP_415218
	Meleagris gallopavo_CA15	XP_003211059.1
	Taeniopygia guttata_CA15	XP_002195938.2
	Anolis carolinesis_CA15	XP_003225153.1
	Xenopus tropicalis_CA15	XP_002931952.2
CA 6	Homo sapiens_CA6	NP_001206.2
	Macaca mulatta_CA6	XP_001099188.2
	Bos taurus_CA6	DAA21262.1
	Equus caballus_CA6	NP_001137421.2
	Mus musculus_CA6	NP_033932.2
	Rattus norvegicus_CA6	NP_001128313.1
	Gallus gallus_CA6	XP_425745.3
	Meleagris gallopavo_CA6	XP_003212306.1
	Taeniopygia guttata_CA6	XP_002187446.1
	Xenopus laevis_CA6	NP_001123372.1
	Xenopus tropicalis_CA6	NP_001085550.1
	Danio rerio_CA6	XP_002666525.1
	Homo sapiens_CA9	NP_001207.2
	Macaca mulatta_CA9	XP_001088481.1
	Equus caballus_CA9	XP_001504561.3

CA 9	Bos taurus_CA9	XP_002689744.1
	Mus musculus_CA9	AAI20545.11
	Rattus norvegicus_CA9	EDL98746.1
	Gallus gallus_CA9	XP_001233320.2
	Danio rerio_CA9	XP_694982.2
CA 12	Homo sapiens_CA12	NP_001209.1
	Macaca mulatta_CA12	EHH27385.1
	Mus musculus_CA12	AAH35941.1
	Rattus norvegicus_CA12	NP_001074225.1
	Bos taurus_CA12	XP_870878.3
	Gallus gallus_CA12	XP_413756.2
	Meleagris gallopavo_CA12	XP_003209429.1
	Xenopus tropicalis_CA12	CAJ81489.1
	Xenopus laevis_CA12	NP_001091227.1
CA 14	Homo sapiens_CA14	BAA85002.1
	Macaca mulatta_CA14	NP_001244832.1
	Equus caballus_CA14	XP_001489157.1
	Bos taurus_CA14	NP_001179134.1
	Mus musculus_CA14	NP_035927.1
	Rattus norvegicus_CA14	NP_001103125.1
	Gallus gallus_CA14	XP_003642713.1
	Meleagris gallopavo_CA14	XP_003212828.1
	Xenopus tropicalis_CA14	NP_001103521.1
	Danio rerio_CA14	NP_001032782.1
	Oreochromis niloticus_CA14	XP_005810348.1
Daphnia CA 5/6/7	Daphnia pulex_CA6B	EFX88008.1
	Daphnia galeata_CA6B	unpublished
	Daphnia pulex_CA6E	EFX88011.1
	Daphnia galeata_CA6E	unpublished
	Daphnia pulex_CA6F	EFX88012.1
	Daphnia galeata_CA6F	unpublished
	Daphnia pulex_CA6C	EFX88009.1
	Daphnia galeata_CA6C	unpublished
	Daphnia pulex_CA6D	EFX88010.1
	Daphnia pulex_CA6G	EFX88013.1
	Daphnia pulicaria_CA5	unpublished
	Daphnia pulex_CA5	EFX81683.1
	Daphnia galeata_CA5	unpublished
	Daphnia pulex_CA6A	EFX88007.1
	Daphnia galeata_CA6A	unpublished
	Daphnia pulex_CA7D	EFX88103.1
	Daphnia galeata_CA7D	unpublished
	Daphnia pulex_CA7A	EFX88100.1
	Daphnia galeata_CA7A	unpublished
	Daphnia pulex_CA7B	EFX880101.1
	Daphnia galeata_CA7B	unpublished
	Daphnia pulex_CA7I	EFX88114.1
	Daphnia pulex_CA7M	EFX88119.1
	Daphnia galeata_CA7I	unpublished
	Daphnia galeata_CA7G	unpublished
	Daphnia pulex_CA7H	EFX88113.1
	Daphnia pulex_CA7L	EFX88118.1
	Daphnia pulex_CA7K	EFX88116.1
	Daphnia pulex_CA7O	EFX88121.1
	Daphnia pulex_CA7P	EFX88123.1
	Daphnia galeata_CA7P	unpublished
	Daphnia pulex_CA7J	EFX88115.1
	Daphnia pulex_CA7N	EFX88120.1
	Daphnia pulex_CA7Q	EFX77528.1
	Daphnia galeata_CA7H	unpublished
	Daphnia galeata_CA7J	unpublished
	Daphnia pulex_CA7C	EFX88102.1
	Daphnia pulex_CA7E	EFX88104.1

	Daphnia galeata_CA7C	unpublished
	Daphnia pulex_CA6H	EFX88180.1
Invertebrate GPI-anchored CA	Callinectes sapidus_gCA	ABN51214.1
	Portunus trituberculatus_gCA	AFV46145.1
	Carcinus maenus_gCA	ABX71209.1
	Branchiostoma floridae_gCA	XP_002601262.1
	Stronglocentrotus purpuratus_gCA	XP_796525.1
	Anopheles gambiae_gCA	ACS28257.1
	Danaus plexippus_gCA	EHJ70026.1
	Patella vulgata_gCA	CCJ509593.1
Bacteria CA	Neisseria gonorrhoeae_CA	YP_207719.1
	Kingella denitrificans_CA	WP_003782874.1
	Pastuerella multocida_CA	WP_005752370.1
	Streptococcus mutans_CA	WP_002278508.1
	Thiorhodococcus drewsii_CA	WP_007041651.1
	Aeromonas caviae_CA	WP_010675444.1
	Yersinia enterocolotica_CA	CCQ39919.1
	Serratia sp_CA	YP_006027343.1
	Enterobacter cloacae_CA	EUL61977.1
	Klebsiella oxytoca_CA	EHS90795.1
	Pantoea vagans_CA	WP_13358087.1
	Thermovibrio ammonificans_CA	YP_004152175.1

Table S2 Listing of conserved amino acid residues for each group of α-CAs along with *Daphnia* α-CAs. The gray highlighted residues represent conserved sites throughout the phylogeny. Dark green represents sites conserved in all extracellular α-CAs, medium green the GPI-anchored CAs, light green the transmembrane CAs, pink the secretory CAs. The dark blue represents sites that are conserved in all intracellular α-CAs, medium blue the cytosolic CAs and red the CA-RPs. The bacterial group shows conserved sites across α-CAs. AS = active site, ZB = zinc-binding site, SB = substrate-binding site, GPI = purported GPI-anchoring site, SuB = disulfide bond site.

	Residue	180	181	182	183	184	185	191	192	193	194	195	196	197	198	199	200	201	202	203	204	205	206	207	212	213	214 SuB	215	216	217	218
Extracellular CAs	GPI-anchored CAs	W		Y									P			W											C				
	Invert GPI CAs	W		Y					G				P			W											C				
	Daphnia CA5	W		Y									P			W											C				
	Daphnia CA6s							W								W											C	G	G		
	Daphnia CA7s															W		E	H						S		C		G		H
	Vert GPI CAs	W	C	Y			Q				C		P			W											C				
	Secretory CAs (CA6s)	W	T	Y					G		L	D	E		H	W				Y							C		G		
	Transmembrane CAs				G				G			G				W									P		C	G	G		
Intracellular CAs	CA-RP												P		F	W	G	L		N			W						G		R
	CA-RP 8									Y		G	V		E	W	G	L		F	P	D					A	N	G	Y	
	CA-RP 11												P		F	W	G	L		N			W				C		G		R
	Daphnia CA3												P	A	F	W	G	L	I	N	P	E	W	S	M	C	N	R	G	R	R
	Daphnia CA4												P	S	F	W	G	L	I	N	P	Q	W	M	L	C	N	K	G	R	R
	CA-RP 10											V	P	S	F	W	G	L	V	N	S	A	W	N	L	C	S	V	G	K	R
	Cytosolic CAs												P			W											A		G		R
	Invert cCAs											G	P			W											A		G		R
	Daphnia CA1												P	H	T	W		A	E	H	Y		E				A	A	G	E	R
	Daphnia CA2										C	G	P	H	S	Y	T	A	K	H	F	L	H				A	P	G	H	R
	Vert cCAs												P			W													G		
	Bacertial CAs	W		Y		G							P			W											C		G		

	Residue	219	220	221	222	223	224	225	226	227	228	229	230	231	232	233	234	235	236	237	238	239	297	298	299	300	301	302	303	304
Extracellular CAs	GPI-anchored CAs	Q	S	P	I	N	I											L								Y				
	Invert GPI CAs	Q	S	P	I	N	I																			Y				
	Daphnia CA5	Q	S	P	I	D	I											F								Y	D			
	Daphnia CA6s	Q	S	P	I		I			P				V					P		F		F		N	Y	G			
	Daphnia CA7s	Q	S	P	I	N												Y	P		F		F			Y				F
	Vert GPI CAs	Q	S	P		N	I											L					F			Y				
	Secretory CAs (CA6s)	Q	S	P	I	D		Q	R		K	V			N			L			L		L		G	Y				
	Transmembrane CAs	Q	S	P		D												L		P					G	Y				
Intracellular CAs	CA-RP	Q	S	P		N									D	P		L												
	CA-RP 8	Q	S	P	I	N	L	N	S	R	E	A		Y	D	P	S	L	L		V	R	L	S	P	N	Y	V		
	CA-RP 11	Q	S	P									L		D	P														
	Daphnia CA3	Q	S	P	I	N	I	E	P	S	Q	L	L	F	D	P	N	L	R	P	L				H	I	D	K	N	A
	Daphnia CA4	Q	S	P	I	D	I	E	P		K	M	L	Y	D	P	N	L	R	P	L				S	V	D	K	H	K
	CA-RP 10	Q	S	P	V	N	I	E	T	S	H	M	I	F	D	P	F	L	T	P	L				R	I	N	T	G	G
	Cytosolic CAs	Q	S	P	I		I								D	P		L												
	Invert cCAs	Q	S	P		D	I								D	P		L												
	Daphnia CA1	Q	S	P	I	D	I	Q	T	S		V	T	L	D	P	K	L	S	S	R	Q	L	T	W	N	W			
	Daphnia CA2	Q	S	P	I	N	I	R	P	D	E	V	K	L	D	P	R	L	N	K	R		L	T	W		Y			
	Vert cCAs	Q	S	P	I		I								D	P		L		P	L				Y	D				
	Bacertial CAs	Q	S	P			I											L												

	Residue	305	306	307	308	309	310	311	312	313	314	315	316 AS	317	318	319	320	321	322	323	324	325	326	327	328	329	334	335	336	337	338	339
Extracellular CAs	GPI-anchored CAs									N	N	G	H			V																
	Invert GPI CAs									N	N	G	H																			
	Daphnia CA5									N	N	G	H			V																
	Daphnia CA6s					M				N	N	G	H							L					P				P		I	T
	Daphnia CA7s	P				E		L		N	N	G	H	T		V		L		I								L	P		I	
	Vert GPI CAs									N		G	H			V															I	
	Secretory CAs (CA6s)		G			F		M		N	N	G	H			V	Q	I		L	P									T	M	
	Transmembrane CAs							L		N	N	G	H			V		L		L	P											
Intracellular CAs	CA-RP					G				N	T	G									K									V	N	
	CA-RP 8	V	C	R	D	C	V			N	D	G	H			I	Q			L	K	S			K	S				V	L	
	CA-RP 11					G		L		N	T	G					F													V	N	
	Daphnia CA3			V	A	G	Y	L	S	N	M	G	Q	G	L	I	F	T	V	D	N	S	S	K				P	S	I	N	L
	Daphnia CA4			V	S	G	V	L		N	T	G	Q	S	L	V	F	R	P	E	T	G	P	K	H		S	L	A	V	N	V
	CA-RP 10	R	K	V	S	G	T	M	Y	N	T	G	R	H	V	S	L	R	L	D	K	E	H	L						V	N	I
	Cytosolic CAs									N		G			W																L	
	Invert cCAs									N		G			W																L	
	Daphnia CA1	P	K	H	T	V	D	V	T	N	T	G	Y	C	W	K	A	H		V	H	G	E	E	S					S	L	E
	Daphnia CA2	P	K	E	T		E	L	L	N	T	G	Y	C	W	K	A	H		V	P	T	E	G	A					T	L	S
	Vert cCAs									N	N	G		S			V		F		D				D	S						
	Bacertial CAs									N	N	G	H	T																		

	Residue		341	342		344	345	346	347	348	349	350	351	350	353 AS	354	355 ZB	356	357 ZB	358	359	360	361	362	363	367	368	369	370
Extracellular CAs	GPI-anchored CAs	G	G	G	L						Y				Q		H	F	H	W	G								G
	Invert GPI CAs	G	G	G	L						Y				Q		H	F	H	W	G								G
	Daphnia CA5	D	G	G	L						F				Q		H	F	H	W	G								G
	Daphnia CA6s	G	G	G	L						Y		F		Q		H		H	W	G		D	S					G
	Daphnia CA7s	G	G	G	L					R	Y		F		Q	L	H	F	H	W	G								G
	Vert GPI CAs	G	G	G	L						Y		A		Q		H		H	W	G								G
	Secretory CAs (CA6s)										Y		A		Q	M	H		H	W	G	G				E		S	G
	Transmembrane CAs										Y		A		Q	L	H	L	H	W	G								G
Intracellular CAs	CA-RP		G	G	P										E				H		G		E						G
	CA-RP 8	G	G	P	L	P			G	H	E	F	E	L		V	R	F	H	W	G	R	E	N	Q			R	G
	CA-RP 11		G	G	P					L	Y				E				H		G								G
	Daphnia CA3	T	G	G	P		L	S	Y	K	Y	E	W	H	Q	M	A	L	H	F	G	L	N	N			Q	S	G
	Daphnia CA4	S	A	G	P		L	A	Y	R	Y	Q	I	E	E	V	F	L	H	Y	G	T	D	N			S	H	G
	CA-RP 10	S	G	G	P		M	T	Y	S	H	R	L	E	E	I	R	L	H	F	G	S	E	D			S	Q	G
	Cytosolic CAs		G	G	P	L					Y		L		Q	F	H		H	W	G								G
	Invert cCAs		G	G	P	L					Y		L		Q		H		H	W	G								G
	Daphnia CA1		G	G	P	L	K	D		K	Y	Q	L	E	Q	Y	H	C	H	W	G	V	N		N			V	G
	Daphnia CA2		G	G	P	L	S	H			Y		L	A	Q	Y	H	C	H	W	G	E	N	D				I	G
	Vert cCAs		G	G	P	L					Y	R	L		Q	F	H	F	H	W	G								G
	Bacertial CAs		G										L		Q		H	F	H		P								

	Residue	371	372 AS	373	374	375	376	377	378	379	380	381	382	383	384	385 ZB	386	387	388	389	390	391	392	393	394	395	396	397	398	399
Extracellular CAs	GPI-anchored CAs	S	E	H			D	G					M	E	L	H		V	H		N			K	Y	G	S	L	G	A
	Invert GPI CAs	S	E	H	T							P	M	E	L	H		V	H	Y					Y	G	S	L	G	A
	Daphnia CA5	S	E	H	T		N	N				A	A	E	L	H	F	V	H	F				K	Y	G		L	G	A
	Daphnia CA6s	S	E	H		I		S	K			P		E	L	H		V	H		N			K	Y	G		F	A	
	Daphnia CA7s	S	E	H		I					Y		A	E		H	I	V	H	Y	N			K	Y	G		F		
	Vert GPI CAs	S	E	H			D	G					M	E		H		V	H		N			K			S			
	Secretory CAs (CA6s)	S	E	H	T		D	G		R				E		H		V	H	Y	N	S		K	Y		S		E	
	Transmembrane CAs	S	E	H				G					A	E		H		V	H	Y	N			K	Y					
Intracellular CAs	CA-RP	S	E	H			N	G		A	F	P		E		Q	L		H		N			L	Y		N			E
	CA-RP 8	S	E	H	T	V	N	F	K	A	F	P	M	E	L	H	L	I	H	W	N	S	T	L			S	I	D	E
	CA-RP 11	S	E	H							F	P	A	E		Q	L				N			L	Y		N		S	
	Daphnia CA3	S	E	H	M	I	A	G	R	A	F	P	A	E	L	Q	V	F	G	F	N	T	Q	L	Y		S	T	Y	E
	Daphnia CA4	S	E	H	R	I	Q	G		A	F	P	A	E	I	Q	F	Y	G	Y	N	A	E	L	Y	A	N	A	S	E
	CA-RP 10	S	E	H	L	L	N	G	Q	A	F	S	G	E	V	Q	L	I	H	Y	N	H	E	L	Y	T	N	V	T	E
	Cytosolic CAs	S	E	H		V	D	G			Y			E	L	H	L	V	H	W	N			K	Y		S	F		E
	Invert cCAs	S	E	H		V	D	G			Y			E	L	H	L	V	H	W	N			K	Y		S	F		E
	Daphnia CA1	S	E	H	L	V	D	G	K	A	Y	A		E		H	F	V	H	W	N		T	K	Y	G	S	F	S	E
	Daphnia CA2	S	E	H	V	V	N	G	Q		Y	G	A	E	I	H	F	V	H	W	N		T	K	Y	G	S	F	N	E
	Vert cCAs	S	E	H	T	V	D	G			Y		A	E	L	H	L	V	H	W	N			K	Y			F		
	Bacertial CAs	S	E	N				G				P		E		H		V	H											

	Residue	400	401	402	403	404	405	407	408	409	410	411	412	413	414	415	416	417	418	419	420	421	422	433	434	435	436	437	438	439	440
Extracellular CAs	GPI-anchored CAs							D		L	A	V	L	G				E		S			D		N	P	A				
	Invert GPI CAs							D	G	L	A	V	L	G				E		S			D		N	P	A				
	Daphnia CA5							D	G	L	A	V	L	G				E		S			D		N	P	A				
	Daphnia CA6s	A						D	G	L	A	V	L										D		N				F		
	Daphnia CA7s	A				H		D	G	L	A	V	L	G	I		I	E	L	Q		R	D		N	I	A		F	R	H
	Vert GPI CAs								G	L	A	V	L												N						
	Secretory CAs (CA6s)	A		D		P			G	L	A	V	L	A										E	N	N	Y	Y	S		F
	Transmembrane CAs	A							G	L	A	V	L					E		G					N			Y			
Intracellular CAs	CA-RP	A							G							F															
	CA-RP 8	A		G	K		H		G	I		I	I	A	L	F	V	Q	I	G	K	E			H		G		L	K	A
	CA-RP 11	A							G					S																	
	Daphnia CA3	A	L	E	S	P	H		G	I	V	A	L	S	V	L	I	Q	I	G	D	A	S	L	Q	T	T	E	F	R	L
	Daphnia CA4	A	R	H	K	S	Q		G	L	V	A		A	V	M		Q	I	G	E	T			P	N		E	L	R	S
	CA-RP 10	A	A	K	S	P	N		G	L	V	V	V	S	I	F		K	V	S		S	S	N	P	F	L	N	R	M	L
	Cytosolic CAs	A							G	L	A	V	L	G	V	F				G							E				
	Invert cCAs	A							G	L	A	V	L	G		F			V	G					H		E				K
	Daphnia CA1	A	L	K	H	C	G		G	L	A	V	L	G	V	F	V		E	G	K	E			H		E		A	N	N
	Daphnia CA2	A	L	K	Y	G	D		G	L	A	V	L	G	V	F	L	K	V	G	K	E			N	P	E		L	N	K
	Vert cCAs	A					D		G	L	A	V		G	V	F	L			G										Q	
	Bacertial CAs									L	A	V	L							G					N						

	Residue	441	442	443	444	445	446	447 GPI	448	449	450	451	452	453	454	455	456	457	460	459	460	461
GPI-anchored CAs	Extracellular CAs					L		N	I													
Invert GPI CAs						L		N	V													
Daphnia CA5						L		N	V													
Daphnia CA6s					Q			E								D	E	T				
Daphnia CA7s		L	E		Q	F	D		I			P								L		
Vert GPI CAs						L		N	I													
Secretory CAs (CA6s)		I	S			L			I				G	Q		T		L			L	
Transmembrane CAs		I			H	L			I											P		F
CA-RP	Intracellular CAs								I		Y	K										
CA-RP 8		V	T	E	I	L	Q	D	I	Q	Y	K	G	K	S	K	T	I	P	C		
CA-RP 11											Y											
Daphnia CA3		L	M	E	H	I	T	K		E	R	P	G						L	L	Q	
Daphnia CA4		L	S	S	L	F	G	Q	V	I	Y	R	G									
CA-RP 10		N	R	D	T	I	T	R	I	T	Y	K	N									
Cytosolic CAs						L			I			K						F				
Invert cCAs																						
Daphnia CA1		L	I		A	L		N	I	R	F	K	D	E	T	V	K	F		D	H	L
Daphnia CA2		L	C		E	L	I	N		K	H	K	D	E	C			F	R			
Vert cCAs				D		L			I			K						F				
Bacertial CAs						L			W				P									

	Residue	469	470	471	472	473	474	475	476	477	478	479	480	481	482	483	484	485	486	487	488	489	490	491	492	493	494	495	496	497	498 AS/SB	499 SB	500	501	502 SuB	503	504	505	506	507
GPI-anchored CAs	Extracellular CAs														L	L	P						F	Y	R	Y		G	S	L	T	T	P		C		E		V	
Invert GPI CAs																L	P						F	Y	R	Y		G	S	L	T	T	P		C		E		V	
Daphnia CA5			L													L	P						F	Y	R	Y		G	S	L	T	T	P		C		E		V	
Daphnia CA6s												L		D		L	P					S	F	Y	R	Y		G	S		T	T	P		C				V	
Daphnia CA7s		S	V	P								L		D	L	L	P			T		S	F	F	R	Y	N	G	S	L	T				C	N	E	D	V	I
Vert GPI CAs															L	L	P			L			Y		R	Y		G	S	L	T	T	P		C				V	
Secretory CAs (CA6s)															M	L	P			L		H		Y		Y		G	S	L	T	T	P	P	C		E		V	
Transmembrane CAs															L	L	P			L			Y		R	Y		G	S	L	T	T	P	P	C				V	
CA-RP	Intracellular CAs											N			L										T	Y		G	S		T		P	P	C		E	T		T
CA-RP 8									F	N	P	N		L	L	P	D	P	L	L	R	D	Y	W	V	Y	E	G	S	L	T		P	P	C	S	E	G	V	T
CA-RP 11												S			L		P		T						T	Y		G	S		T		P		C		E	T		T
Daphnia CA3		P	G	Q	E	R	P	M	P	A	L	S	L	K	M	L	L	P	P		D	Y	Y	I	T	Y	E	G	S	A	T	T	P	A	C	Q	E	T	S	T
Daphnia CA4				Q	S	A	D	L	R	H	V	S	L	H	A	L	L	P	E	T	D	H	Y	M	T	Y	E	G	S	T	T	H	P	G	C	W	E	T	T	S
CA-RP 10				D	A	Y	L	L	Q	G	L	N	I	E	E	L	Y	P	E	T	S	S	F	I	T	Y	D	G	S	M	T	I	P	P	C	Y	E	T	A	S
Cytosolic CAs										D	P				L								Y	W	T	Y		G	S	L	T	T	P	P	C		E	S	V	
Invert cCAs											P				L	V							Y	W	T	Y		G	S	L	T	T	P		C		E	S	V	
Daphnia CA1								E	L	D	S	K	L	M	P			N	E	P	R	S	Y	W	T	Y	L	G	S	L	T	T	P	P	C	S	E	C	V	I
Daphnia CA2									L	D	P	N	K	L	L	P					G	A	Y	Y	T	Y	L	G	S	L	T	T	P	P	C	N	E	C	V	I
Vert cCAs									F	D	P			L	L	P						D	Y	W	T	Y		G	S	L	T	T	P	P	L		E	S	V	T
Bacertial CAs															L		P						Y	Y	R	F		G	S	L	T	T	P	P	C		E	G	V	

	Residue	508	509	510	511	512	513	514	515	516	517	518	519	520	521	522	532	533	534	535	536
GPI-anchored CAs	Extracellular CAs	W	T	V	F				I		I	S			Q	L				F	R
Invert GPI CAs		W		V	F				I		I	S			Q	L				F	R
Daphnia CA5		W	T	V	F				I			S			Q	L				F	R
Daphnia CA6s		W	T		F	D						S	E		Q					F	R
Daphnia CA7s		W	T		F		T	P	I	A	I	S	E	R	Q	L			K	F	R
Vert GPI Cas		W	T		F						I				Q					F	
Secretory CAs (CA6s)		W		V							L	S			Q					L	E
Transmembrane CAs		W	T	V	F				V		I	S			Q	L				L	
CA-RP	Intracellular CAs	W	I		F			P			I	T			Q					L	R
CA-RP 8		W	I	L	F	R	Y	P	L	T		S	Q		Q		I	E	E	F	R
CA-RP 11		W	I					P			I	T								L	R
Daphnia CA3		W	I		F	N	R	P	L	Y	I	T	E	Q	Q		L	Y	A	L	R
Daphnia CA4		W	I	I	Y	N	K	P	I	Y	I	T	R	Q	E		L	Y	A	L	R
CA-RP 10		W	I	I	M	N	K	P	V	Y	I	T	R	M	Q		M	H	S	L	R
Cytosolic CAs		W	I	V	F			P	I		V	S			Q		L			F	R
Invert cCAs		W	I	V	F			P	I		V	S		E	Q		L				R
Daphnia CA1		W	I	V	Y	K		P	I	Q	M	S	H		Q		L	E	A	F	R
Daphnia CA2		W	I	V	F	V	E	P		E	V	S	H	E	W		L	E	S	F	R
Vert cCAs		W	I			K		P	I		V	S			Q					F	R
Bacertial CAs		W		V		K						S			Q					F	

	Residue	537	538	539	540	541	542	543	544	545	546	547	548	549	550	551	552	553	554	555	556	557	559	560	561	562	563	564	565	566	567	568	569	570	571	572	573	574	575	576
GPI-anchored CAs	Extracellular CAs																																	N		R	P		Q	P
Invert GPI CAs																																		N		R	P		Q	P
Daphnia CA5																																	N	N		R	P		Q	P
Daphnia CA6s				L																														N		R	P		Q	P
Daphnia CA7s				L		D			G																						L			N		R	P		Q	
Vert GPI Cas																																		N		R	P		Q	P
Secretory CAs (CA6s)		N		L		D			N																					T				N	Y	R			Q	P
Transmembrane CAs				L																														N		R			Q	P
CA-RP	Intracellular CAs		L	L	Q																												D	N	F	R	P		Q	P
CA-RP 8		R		L	R	T	H	V	K	G				A	E	L		E	G	C	D								G	I	L	G	D	N	F	R	P	T	Q	P
CA-RP 11			L		Q																										L			N		R	P		Q	P
Daphnia CA3		G	L	K	R	G	S	P	N	P	N	H	N	Q	I	K	S	S	S	F	S	S	S	L	S	V			D	P	M	G	N	N	Y	R	P	I	Q	P
Daphnia CA4		R	L	V	Q	G	D	S																	E	T	P	K	G	A	L	G	N	N	V	R	A	L	Q	P
CA-RP 10		L	L	S	Q	N	Q	P	S																	Q	I	F	L	S	M	S	D	N	F	R	P	V	Q	P
Cytosolic CAs																																		N		R	P			P
Invert cCAs																																		N		R	P			P
Daphnia CA1		A	M	Y	C	G	D	K	E	Q	C	C	A	A	N	E	M	A	E	C											I	L	T	N	Y	R	P	C	L	P
Daphnia CA2		E	M	M	S	M	K	K	G	G	C	S	Q	P		S	R	E	H															N		R	P		Q	S
Vert cCAs			T	L	L				E							K																				R	P		Q	P
Bacertial CAs																																		N	N	R	P		Q	P

	Residue	577	578	579	580	581	582	583	584	585	586	587	588	589	590	591
GPI-anchored CAs	Extracellular CAs	L			R		V			S						
Invert GPI CAs		L			R		V			S						
Daphnia CA5		L			R		V			R						
Daphnia CA6s					R				Y	R						
Daphnia CA7s		L	H		R		V		Y							
Vert GPI Cas					R					S						
Secretory CAs (CA6s)		L			R	V	V	E			F					
Transmembrane CAs					R					S	F					
CA-RP	Intracellular CAs	L			R			R		N						
CA-RP 8		L	S	D	R	V	I	R	A	A	F	Q				
CA-RP 11		L			R			R		N						
Daphnia CA3		T	H	H	R	P	V	R	T	N	I	D	F	T	S	S
Daphnia CA4		L	H	H	R	T	V	R	T	N	I	D	F	T	N	
CA-RP 10		L	N	N	R	C	I	R	T	N	I	N	F	S	L	Q
Cytosolic CAs					R		V	R			F					
Invert cCAs					R		V	R								
Daphnia CA1			G	E	R	C	V	R	A							
Daphnia CA2		L			R		V	R		S						
Vert cCAs		L			R		V	R			F					

Table S3 Ancestral states of amino acids at informative residues. Ancestral states were inferred using a Maximum Likelihood model in MEGA 5.0 and the most probable states at a given residue were selected. CA groups are based on the most recent common ancestor as determine by the phylogenic analysis, for example the hypothetical ancestral Animalia α-CA state is the predicted sequence of the most recent common ancestor of the extracellular α-CAs and intracellular α-CAs, while the extracellular α-CA hypothetical ancestor is the most recent common ancestor to the invertebrate and vertebrate clades of Extracellular α-CAs (Figure1). The residues are based on the alignment performed for phylogeny construction. Codes on the residues are as follows: SuB = disulfide bond site, AS = active site, ZB = zinc-binding site, and SB = substrate-binding site. Shaded amino acids reflect an amino acid change from the most common recent ancestor and the shaded amino acids in a box are amino acid change that resulted in convergent evolution. The no. of amino acid changes reflects the number of changes from the most common recent ancestor for that group.

Residue / CA Group	214 SuB	232	233	314	316 AS	318	319	351	353 AS	354	355 ZB	356	381	385 ZB	387	419	434	445	448	491	492	499 SB	501	505	512	573	578	No. of Amino Acid Changes
Bacterial	C	P	I	N	H	I	Q	L	Q	F	H	F	P	H	V	G	N	L	W	Y	R	T	P	G	K	P	N	
Ancestral Animalia CA	C	D	I	N	H	I	Q	L	Q	F	H	F	P	H	V	G	N	L	I	Y	R	T	P	G	K	P	N	2
Extracellular	C	D	I	N	H	V	D	L	Q	F	H	F	P	H	V	S	N	Y	I	Y	R	T	P	V	K	P	N	5
Invertebrate	C	D	I	N	H	V	T	A	Q	L	H	F	P	H	V	S	N	F	I	Y	R	T	T	V	E	P	N	6
Vertebrate	C	D	S	N	H	V	D	A	Q	F	H	F	P	H	V	S	N	Y	I	Y	R	T	P	V	E	P	N	3
GPI	C	D	S	N	H	V	D	A	Q	F	H	F	P	H	V	S	N	Y	I	Y	R	T	P	V	E	P	N	0
CA 4	C	D	S	N	H	V	D	A	Q	F	H	F	P	H	V	S	N	Y	I	Y	R	T	P	T	E	P	N	1
CA 15	C	D	S	D	H	L	S	A	Q	F	H	F	P	H	V	S	N	Y	V	Y	R	T	D	V	E	P	K	6
Secretory																												
CA6	C	D	P	N	H	I	S	A	Q	M	H	L	V	H	V	G	E	Y	I	Y	R	T	P	S	D	A	N	11
Transmembrane	C	D	P	N	H	L	S	A	Q	L	H	L	P	H	V	G	N	Y	L	Y	R	T	P	S	N	A	N	10
CA9	C	S	P	N	H	L	T	A	Q	L	H	L	P	H	V	G	N	Y	L	F	R	T	P	S	N	A	N	3
CA12	C	D	S	N	H	M	S	A	Q	L	H	L	A	H	V	G	N	Y	I	Y	R	T	P	S	R	Q	N	6
CA14	C	D	P	N	H	L	S	A	Q	L	H	L	P	H	V	G	N	Y	L	Y	R	T	P	S	N	A	N	0
Intracellular	C	D	P	T	H	S	F	L	Q	F	H	F	P	H	V	G	N	L	T	W	T	T	P	S	K	P	N	8
CA-RP	C	D	P	T	H	T	V	L	E	I	R	F	P	H	I	G	N	L	T	W	T	T	P	S	K	P	N	6
CA8	N	D	P	I	H	T	V	L	E	V	R	F	P	H	I	G	H	L	T	W	T	T	P	G	R	P	S	7
CA10	C	D	P	R	H	S	D	L	E	I	R	L	S	Q	I	G	N	N	N	I	T	I	P	T	N	P	N	12
CA11	C	D	P	R	S	S	D	L	E	I	R	L	P	Q	I	G	N	L	T	M	T	T	A	T	N	P	H	11
Cytolsolic	A	D	P	T	N	S	F	L	Q	F	H	F	P	H	V	G	H	L	T	W	T	T	P	S	K	P	K	4
Invertebrate	A	D	P	T	T	S	F	L	Q	F	H	A	P	H	V	G	H	L	T	W	T	T	P	S	K	P	K	2
Vertebrate	A	D	P	S	N	S	F	L	Q	F	H	F	P	H	V	G	H	L	T	W	T	T	P	S	K	P	K	1
CA5	G	D	P	W	N	S	F	L	Q	F	H	F	P	H	V	G	H	L	V	W	T	T	P	S	K	P	K	3
CA7	E	D	P	S	H	S	V	L	Q	F	H	F	P	H	V	G	H	L	T	W	T	T	P	S	K	P	K	3
Fish CA1/2	N	D	A	L	H	S	F	L	Q	F	H	F	P	H	V	G	N	L	L	W	T	T	P	S	K	P	K	5
CA1	N	D	P	V	H	S	F	L	Q	F	H	F	A	H	V	G	N	L	L	W	T	H	P	C	K	P	K	4
CA2	N	D	P	V	H	S	F	L	Q	F	H	F	A	H	V	G	K	L	L	W	T	T	P	C	K	P	K	4
CA3	K	D	P	L	N	T	C	L	Q	F	H	F	A	H	V	G	K	M	L	W	T	T	P	C	K	P	K	7
CA13	N	D	P	L	H	S	F	L	Q	F	H	F	A	H	V	G	N	L	T	W	T	V	P	S	K	P	K	3

Figure S1. Phylogeny of α-CAs inferred from a maximum-likelihood analysis performed with RaxML version 8.0 with 1000 iterations. Bootstrap values are indicated at the nodes. Species are collapsed within a larger taxonomical grouping.

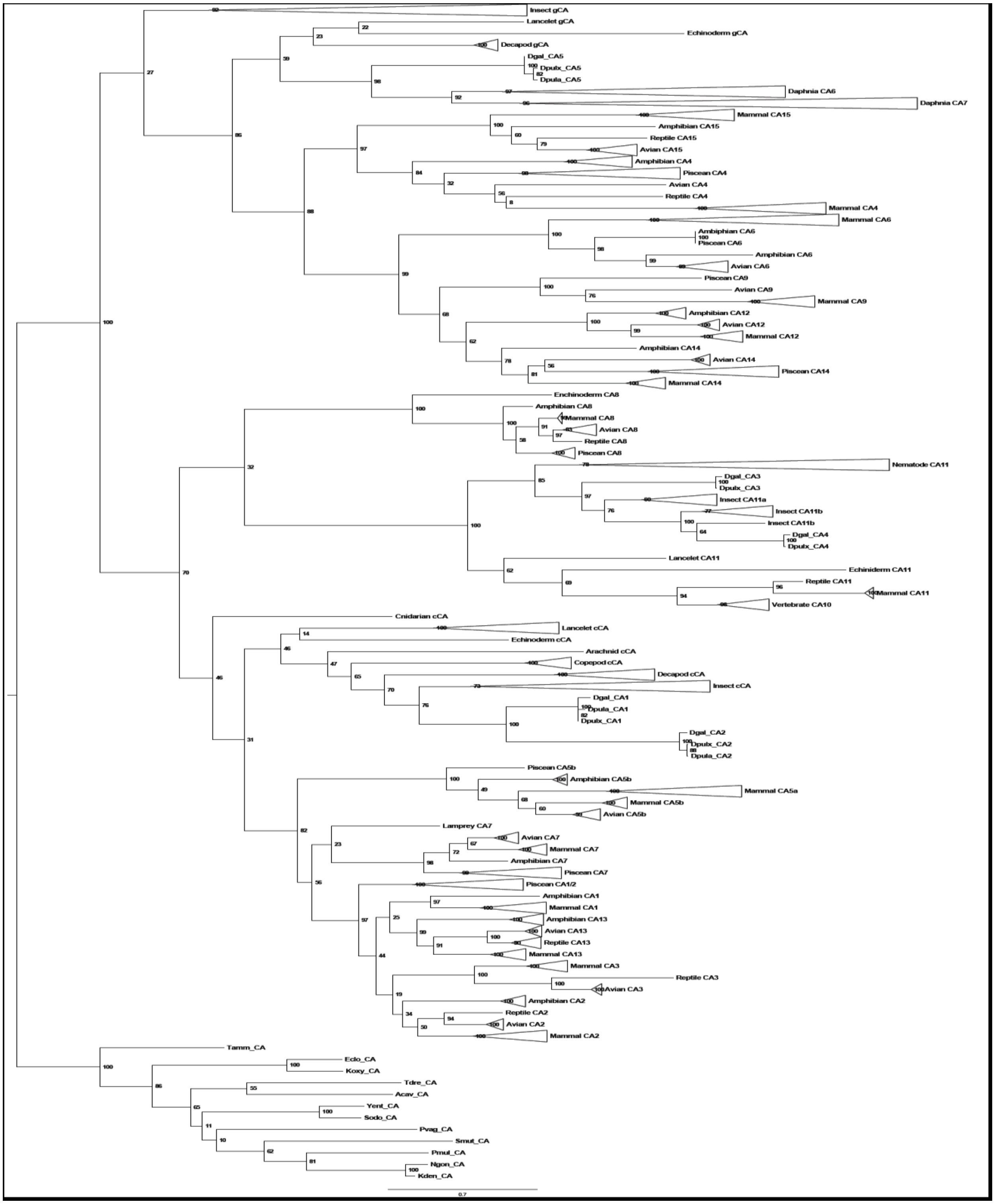

Supplement: Supplementary file 1 — The supplementary material that follows includes the following tables and figures: Tables S1, S2, S3, and Figure S1. We constructed Table S1 to list the taxa with their respective isoforms of α-Carbonic Anhydrase (CA) that were used in both the MrBayes and RaxML phylogenetic analysis. Since, branches of the resultant phylogenies are collapsed for clarification of the figures, it was necessary to provide a list of the taxa used. We also provide the NCBI ascension number of the CAs used. Table S2 is listing of the conserved amino acid residues among the α-CA isoforms and taxonomical groupings. It was compiled using the alignment of the amino acid sequences (see Material and Methods). Residues that are conserved within α-CA groupings are highlighted by a color based upon which grouping they are associated (see Table legend). Table S3 list the ancestral states of informative amino acid residues (see Materials and Methods for criteria) based upon the phylogeny. Each node of the phylogeny is represented in the table with the predicted ancestral amino acid sequence. The ancestral states were inferred using a maximum likelihood approach. Figure S1 is the result of our RaxML phylogenetic analysis. This tree has a similar topology as the MrBayes tree that is printed in the main manuscript. This tree was run to verify or MrBayes results. The main difference between these two trees is that the RaxML tree resolves the polytomy in the vertebrate cytosolic α-CAs, but with low support. [file 538918.f1.pdf]
